# Nucleic Acids in Cancer Diagnosis and Therapy

**DOI:** 10.3390/cancers15071938

**Published:** 2023-03-23

**Authors:** Taewan Kim

**Affiliations:** 1 Department of Anatomy, Histology and Developmental Biology, International Cancer Center, Shenzhen University School of Medicine, Shenzhen 518055, China; kim.2203@buckeyemail.osu.edu; 2 The Ohio State University Comprehensive Cancer Center, Columbus, OH 43210, USA

Nucleic acids include two main classes: deoxyribonucleic acid (DNA) and ribonucleic acid (RNA). In the last few decades, a cohort of oncogenes and tumor suppressor genes has been discovered at the DNA level. Those genes are abnormally activated or inactivated by the genetic mutations of DNA in cancer cells. As a result, it is known that genetic mutations in DNA play a pivotal role in cancer development [1]. In addition, the highlighting of genetic mutations in cancer has led to translational cancer research using genetically engineered animal models [2]. To date, a number of cancer-associated genes have been effectively targeted cancer treatments [3]. Nonetheless, growing evidence ironically indicates that the pivotal genetic cause is not the sole cause of cancer development. The cancer therapeutic strategies targeting the mutated cancer-associated genes or their proteins have frequently faced difficulties, such as non-response, resistance, and recurrence [3]. It suggests that epigenetic modifications and alterations in conjunction with genetic modifications play a critical role in cancer development.

RNA was considered to be an intermediate messenger between DNA and functional proteins in the central dogma of molecular biology. Therefore, protein-coding mRNAs were centered on RNA biology. Although such non-coding RNAs as ribosomal RNA (rRNA) and transfer RNA (tRNA) were actively studied for their visible functions in protein synthesis, other non-coding RNAs were thought to be byproducts in gene expression in spite of studies implicating non-coding RNAs, such as H19 [4,5]. Since the discovery of microRNA functions in cancer, the role of non-coding RNAs has been highlighted and a wider range of non-coding RNAs has been characterized [6,7,8,9]. Beyond the identification and expression profiling of novel non-coding RNAs in cancer, RNA biology is expanding its research focus, including the modification, bioengineering, delivery, binding partners, and novel functional mechanisms of RNA [10,11,12].

This Special Issue mainly introduces the various findings and current trends of RNA research and their potential in cancer diagnosis and therapy. Fekete et al. show that microRNAs are prognostic markers of platinum-based therapy of squamous cell carcinomas (SCC), such as cervical, head and neck, and lung tumors [13]. Zhang et al. found that miR-125b promoted the migration and invasion of colorectal cancer (CRC) by targeting CFTR and CGN [14]. Along with these research findings, the role of microRNAs in papillary thyroid cancer is introduced in this Special Issue [15]. In addition to microRNAs, the up-to-date discoveries of long non-coding RNAs (lncRNAs) in glioblastoma are covered in this Special Issue [16]. Lung cancer is one of the most common cancer types. As a result, the investigation of non-coding RNAs has been enormously active for several decades. Le et al. comprehensively summarized and introduced various types of non-coding RNAs and their clinical applications in lung cancer [17]. Furthermore, the significant role of recently highlighted non-coding RNAs, including small nucleolar RNAs (snoRNAs) and circular RNAs (circRNAs), were investigated in breast cancer and oral cancer [18,19]. In addition, it is shown in this Special Issue that not only non-coding RNAs but also coding RNAs, including NOTCH1 mRNA, are notable prognostic markers in head and neck squamous cell carcinoma (HNSCC) [20].

With the growing interest in RNA modification, non-coding RNA modifications are also covered in this Special Issue. For instance, microRNA isoforms generated by microRNA modifications, such as A-I RNA editing, bring about racial disparities in lung cancer [21]. The advanced methodology of microRNA modification is also introduced by Marceca et al. [22]. In addition to RNA modifications, RNA-binding proteins were also discussed. Kang et al. reviews and summarizes the enormous amount of information on RNA-binding proteins in cancer [23]. The albumin nanostructure, interacting with siRNAs, plasmids, and antisense oligonucleotides, was also examined as a novel nucleic acid delivery system in cancer treatment [24]. Moreover, a nucleolar protein DKC1 induces rRNA pseudouridylation through snoRNAs and stabilizes telomerase RNA components, thus promoting aggressive mammary cells [25]. Lastly, Wang et al. show the integrative analysis method using an artificial intelligence deep learning technology to detect cancer with non-coding RNAs [26].

This Special Issue deals with a broad spectrum of nucleic acids in cancer, especially focused on various RNA types, modifications, functions, and analytical methodologies with interesting discoveries and summaries of RNA and cancer research. Overall, the research and review articles in this Special Issue provide insight into the current and future directions of RNA research in cancer. Although not discussed in this Special Issue, the critical roles of extracellular RNA, delivered by microvesicles and exosomes, have also been elucidated in other works [27,28,29]. In addition to RNA molecules, novel DNA types, such as extracellular DNA and extrachromosomal DNA, have recently attracted the interest of cancer researchers [30,31]. Furthermore, there is no doubt that DNA and histone modifications are critical regulators of cancer development [1]. Therefore, the novel types of DNA and DNA modifications will be also interesting research subjects in terms of RNA molecules in the epigenetics of cancer research.
